# Successful endoscopic biliary intervention using a rotatable sphincterotome and a multi-ring traction device

**DOI:** 10.1055/a-2760-9422

**Published:** 2025-12-19

**Authors:** Tomohiro Ishii, Kazuya Sugimori, Arisa Omata, Shoichiro Yonei, Hideyuki Anan, Takashi Kurosawa, Shin Maeda

**Affiliations:** 1 89460 Department of Gastroenterology, Saiseikai Yokohamashi Nanbu Hospital, Yokohama, Japan; 2 Department of Gastroenterology, Yokohama City University Graduate School of Medicine, Yokohama, Japan

Biliary cannulation has been reported to be difficult using traction techniques 1 2 or a rotatable sphincterotome 3 ; only few studies have attempted the combined use of these methods. Here, we report a case wherein biliary cannulation was successfully achieved using a rotatable sphincterotome after adjusting the papillary position via a two-step traction technique using a multi-ring traction device.

A 75-year-old woman underwent laparoscopic cholecystectomy for pericholecystic abscess and acute cholecystitis ( Fig. 1 ). Endoscopic retrograde cholangiopancreatography (ERCP) was performed to treat the common bile duct stone identified preoperatively using magnetic resonance cholangiopancreatography ( Fig. 2 ). During the initial ERCP, the presence of a papillary diverticulum necessitated multiple techniques; however, only pancreatic duct cannulation was achieved. Pancreatic stenting was performed after endoscopic pancreatic sphincterotomy. A second ERCP was attempted 2 days later. Despite attempting multiple techniques, biliary cannulation was unsuccessful. Therefore, we attempted biliary cannulation using both a seven-ring traction device (Adachi, Osaka, Japan; Fig. 3 ) and a rotatable sphincterotome (Engetsu; Kaneka Medix, Osaka, Japan). First, the papilla was pulled toward the 7 o’clock position. To visualise the bile duct opening from the front, the loop between the first and second clips was pulled further toward the 4 o’clock position. As the papillary diverticulum did not allow the axis of a standard catheter to align in the bile duct direction, the sphincterotome was rotated to 2 o’clock, enabling successful bile duct cannulation ( Video 1 ). The bile duct stones were successfully removed using endoscopic papillary balloon dilation and the sphincterotome was freely rotated to facilitate loop resection. The traction device used in this case featured seven rings, allowing two-step traction by pulling any ring to secure a better field of view. Thus, the combined use of the device and a rotatable sphincterotome could be an effective approach to achieve bile duct cannulation.

**Fig. 1:**
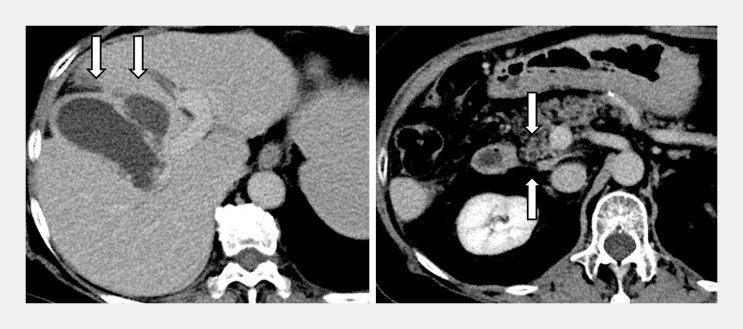
Portal phase of contrast-enhanced computed tomography (CT) showing an enlarged gall bladder and a gall bladder-surrounding abscess (left). The wall thickening of the distal bile duct is observed, but common bile duct stones cannot be identified on CT (right).

**Fig. 2:**
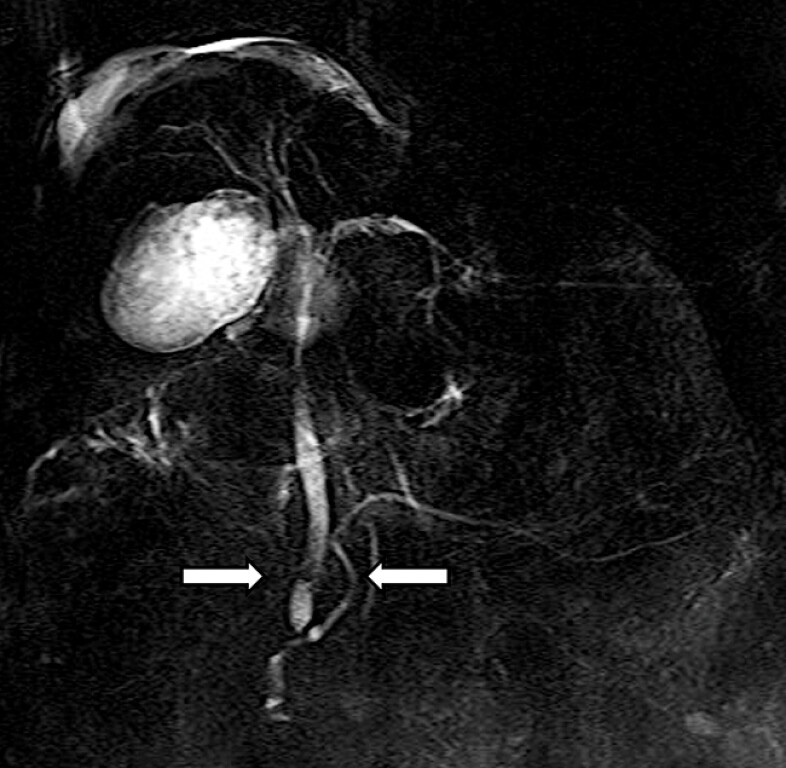
Magnetic resonance cholangiopancreatography reveals a common bile duct stone in the distal bile duct.

**Fig. 3:**
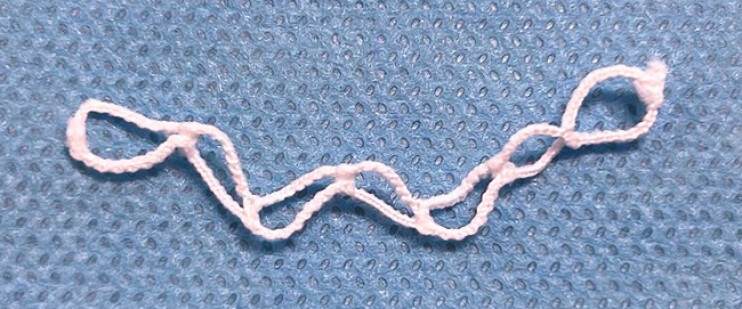
A seven-ring traction device can apply traction through seven elastic loops.

Two-step traction using a multi-rings traction device enables a better view of the bile duct opening.Video 1

Endoscopy_UCTN_Code_TTT_1AR_2AK

## Notes

Endoscopy E-Videos https://eref.thieme.de/e-videos
*E-Videos* is an open access online section of the journal *Endoscopy* , reporting on interesting cases and new techniques in gastroenterological endoscopy. All papers include a high-quality video and are published with a Creative Commons CC-BY license. Endoscopy E-Videos qualify for HINARI discounts and waivers and eligibility is automatically checked during the submission process. We grant 100% waivers to articles whose corresponding authors are based in Group A countries and 50% waivers to those who are based in Group B countries as classified by Research4Life (see: https://www.research4life.org/access/eligibility/ ). This section has its own submission website at https://mc.manuscriptcentral.com/e-videos .
